# Protein expression changes induced in a malignant melanoma cell line by the curcumin analogue compound D6

**DOI:** 10.1186/s12885-016-2362-6

**Published:** 2016-05-18

**Authors:** Marina Pisano, Antonio Palomba, Alessandro Tanca, Daniela Pagnozzi, Sergio Uzzau, Maria Filippa Addis, Maria Antonietta Dettori, Davide Fabbri, Giuseppe Palmieri, Carla Rozzo

**Affiliations:** Institute of Biomolecular Chemistry, National Research Council of Italy, Traversa la Crucca, 3, 07100 Sassari, Italy; Proteomics Laboratory, Porto Conte Ricerche, Tramariglio, Alghero, Italy; Biosistema Scrl, Sassari, Italy

**Keywords:** Melanoma cells, Curcumin, Hydroxylated biphenyls, Proteomic profiling, Molecular pathways analysis

## Abstract

**Background:**

We have previously demonstrated that the hydroxylated biphenyl compound D6 (3E,3′E)-4,4′-(5,5′,6,6′-tetramethoxy-[1,1′-biphenyl]-3,3′-diyl)bis(but-3-en-2-one), a structural analogue of curcumin, exerts a strong antitumor activity on melanoma cells both in vitro and in vivo. Although the mechanism of action of D6 is yet to be clarified, this compound is thought to inhibit cancer cell growth by arresting the cell cycle in G2/M phase, and to induce apoptosis through the mitochondrial intrinsic pathway. To investigate the changes in protein expression induced by exposure of melanoma cells to D6, a differential proteomic study was carried out on D6-treated and untreated primary melanoma LB24Dagi cells.

**Methods:**

Proteins were fractionated by SDS-PAGE and subjected to in gel digestion. The peptide mixtures were analyzed by liquid chromatography coupled with tandem mass spectrometry. Proteins were identified and quantified using database search and spectral counting. Proteomic data were finally uploaded into the Ingenuity Pathway Analysis software to find significantly modulated networks and pathways.

**Results:**

Analysis of the differentially expressed protein profiles revealed the activation of a strong cellular stress response, with overexpression of several HSPs and stimulation of ubiquitin-proteasome pathways. These were accompanied by a decrease of protein synthesis, evidenced by downregulation of proteins involved in mRNA processing and translation. These findings are consistent with our previous results on gene expression profiling in melanoma cells treated with D6.

**Conclusions:**

Our findings confirm that the curcumin analogue D6 triggers a strong stress response in melanoma cells, turning down majority of cell functions and finally driving cells to apoptosis.

**Electronic supplementary material:**

The online version of this article (doi:10.1186/s12885-016-2362-6) contains supplementary material, which is available to authorized users.

## Background

Malignant melanoma (MM) is the most aggressive skin cancer, and its incidence has dramatically risen in all Western countries during the last half century [1]. Although most melanoma cases are early diagnosed and surgically resected, until recently later stages had very poor survival rates because of the lack of effective therapies [2]. In very recent years, several therapeutic approaches - including immune-targeted treatments (anti-CTLA4 agent ipilimumab, anti-PD-1 agent nivolumab, and anti-PD-L1 agents such as lambrolizumab) or inhibitors of key effectors of the MAPK pathway (BRAF-mutant inhibitors as vemurafenib or dabrafenib, MEK inhibitors as cobimetinib, trametinib, and their combination) - are allowing to overcome the ineffectiveness of the conventional therapies and achieve an impressive improvement of the patients’ survival [3, 4]. However, tumor responses produced by the main targeted inhibitors are largely partial and tumor resistance typically develops in few months as a consequence of the activation of alternative proliferation-inducing pathways [5, 6]. Since it is thus unlikely that inhibition of a single component in signaling pathways could yield significantly durable antitumor responses, drug combinations are awaited for a more effective anti-tumor therapy.

Natural products have afforded a rich source of compounds that have found many applications in cancer therapy [7]. Among such products curcumin, a polyphenol extracted from the rhizome of the plant *Curcuma longa,* represents an interesting and promising anticancer therapeutic compound. It is a highly pleiotropic molecule that causes inhibition of proliferation, invasion, angiogenesis, and metastasis in several types of cancer through interaction with multiple cell signaling proteins [8].

**Fig. 1:**
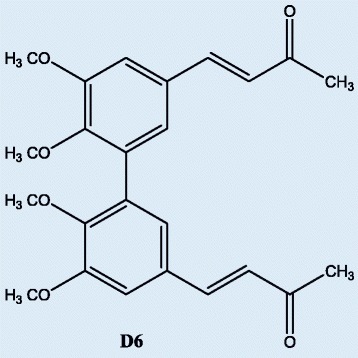
D6 (3E,3′E)-4,4′-(5,5′,6,6′-tetramethoxy-[1,1′-biphenyl]-3,3′-diyl)bis(but-3-en-2-one). Molecular structure

We have previously characterized the antitumor activity exerted by a curcumin analogue called D6 on melanoma cells (Fig. 1). This compound was able to inhibit cell proliferation and induce apoptosis on melanoma cell lines. Tests in vivo showed that D6 could reduce tumor growth on melanoma mice models [9]. We also demonstrated that D6 caused a G2/M arrest of cell cycle and microarrays gene expression profiling of D6 treated melanoma cells showed the presence of important changes in gene expression. Results of this analysis pointed out the induction of strong cell stress responses, with up regulation of several heat shock proteins (HSPs) and involvement of protein ubiquitination and stress response pathways, including p53 driven pathways, strongly supporting the pro-apoptotic activity previously observed. Cell proliferation pathways were instead down-modulated [10].

Proteomic approaches enable an in-depth characterization of global changes occurring at a protein level. One-dimensional polyacrylamide gel electrophoresis is widely used as fractionation step prior to liquid chromatography-tandem mass spectrometry to reduce sample complexity, with remarkable performance in terms of proteome coverage and the added advantage of maintaining information concerning protein molecular weight [11, 12]. Among proteomic quantitation methods, label-free strategies have proven to be more cost-effective, time-saving and flexible compared to labeling techniques, although being less accurate for low-abundance protein [13–15]. The spectral counting approach, in particular, builds on the observation that the number of tandem mass spectra detected in data-dependent acquisition for a given protein are proportional to the protein amount [16].

In order to investigate on the changes induced by D6 at the proteome level and to verify if and to what extent mRNA expression changes relate to protein expression changes, a label-free differential proteomic analysis was carried out on the MM cell line LB24Dagi treated with D6. Results of such analysis are described in this paper.

## Methods

### Reagents

The curcumin analogue D6 (3E,3′E)-4,4′-(5,5′,6,6′-tetramethoxy-[1,1′-biphenyl]-3,3′-diyl)bis(but-3-en-2-one) (Fig. 1) was synthesized in our lab as previously described [9]. For melanoma cell treatment, D6 stored as 100 mM stocks in dimethyl sulfoxide (DMSO) was diluted in complete medium to contain <0.1 % DMSO, immediately before use.

### Cell cultures and D6 treatments

The melanoma cell line LB24Dagi (LB) was kindly provided by the “Istituto Dermopatico dell’Immacolata” in Rome. It was chosen among primary short term cell cultures established from tumor samples of donor patients with proven diagnosis of melanoma, as previously reported [17]. In particular, all primary melanoma cell lines, including the LB one, were generated from surgically-excised tumor tissues, using standard procedures for in vitro isolation and propagation of cells from dissected tumor samples. Briefly, micro-dissections of tumor tissues were incubated with specific growth medium containing gentamicin (50 mcg/ml), fungizone (2.5 mcg/ml), and DNase I (1 U/ml). For all melanoma cell lines, tumor tissues were processed after obtaining the patient’s informed consent and according to the approval by the Ethics Committee at the “Istituto Dermopatico dell’Immacolata” in Rome.

The LB24Dagi MM cells were cultured in RPMI supplemented with 10 % FBS and penicillin/streptomycin [100 IU (50 μg)/ml] in a humidified 5 % CO_2_ atmosphere at 37 °C, either alone or in the presence of 10 μM D6 for 24 h. Cells were then harvested and washed with PBS, and the pellets (10^7^ cells each) were stored at −80 °C pending analyses. The experiment, including both 10 μM D6 treated and untreated cells, was carried out in triplicate, finally obtaining six cell pellets.

### Protein extraction and quantification

Proteins were extracted as follows. Cell pellets were resuspended in the 2-D Protein Extraction Buffer V from GE Healthcare (Little Chalfont, UK; 100 μl buffer per 2.5 × 10^6^ cells) and sequentially subjected to vortexing (two cycles comprising incubation on ice for 5 min followed by vortexing for 30 s), freeze-thawing (two cycles comprising incubation at −80 °C for 15 min followed by thawing at RT for 15 min) and sonication (ten cycles comprising sonication in a Transsonic Digital ultrasonic bath (Elma Electronic, Wetzikon, Switzerland) for 1 min followed by incubation on ice for 30 s). Finally, samples were centrifuged for 15 min at 16,000 × *g* at 4 °C, and the protein containing supernatant was quantified using the Bradford method [18].

### SDS-PAGE, in gel digestion and liquid chromatography-tandem mass spectrometry

Thirty micrograms of each protein extract were separated by SDS-PAGE in 4–20 % TGX gels (Bio-Rad, Hercules, CA, USA) and stained with SimplyBlue SafeStain (Invitrogen, Carlsbad, CA, USA), according to the manufacturer instructions. Then, each whole lane was fractionated into 35 gel slices, which were destained, reduced, carbamidomethylated, and trypsin digested as described previously [19].

LC-MS/MS analyses were performed on a Q-TOF hybrid mass spectrometer equipped with a nano lock Z-spray source, and coupled on-line with a capillary chromatography system CapLC (Waters, Manchester, UK), as described elsewhere [19].

### Data analysis

Proteome Discoverer Mass Informatics Platform (version 1.4; Thermo Scientific) was used for protein/peptide identification, using a workflow consisting of the following nodes (and respective parameters): Spectrum Selector (precursor mass range: 350–5000 Da; S/N Threshold: 1.5), Sequest-HT as search engine (Protein Database: UniProtKB/SwissProt, release 2014_10, taxonomy *Homo sapiens*; Enzyme: Trypsin; Max. missed cleavage sites: 2; Peptide length range 5–50 amino acids; Max. Delta Cn: 0.05; Precursor mass tolerance: 10 ppm; Fragment mass tolerance: 0.02 Da; Static modification: cysteine carbamidomethylation; Dynamic modification: methionine oxidation), and Percolator for peptide validation (FDR <1 %, based on peptide q-value). Protein grouping was allowed according to the maximum parsimony principle.

The Normalized Spectral Abundance Factor (NSAF) was calculated in order to estimate protein abundance [20]. Specifically, a spectral abundance factor (SAF) was obtained by dividing the number of spectral counts (SpCs) of a protein by its number of amino acids [21]; then, NSAF values were obtained by dividing the SAF values by the SAF sum for all proteins identified in each sample [20]. NSAF values were finally multiplied by a scale factor corresponding to the average number of SpCs identified per sample (in order to deal with integers). The beta-binomial test (along with correction for multiple testing) was applied to identify statistically significant variations between sample groups (FDR <0.05). Protein fold-change was calculated by dividing the mean NSAF values for a given protein in one sample group by the mean NSAF values for that given protein in the other sample group, using a correction factor (CF = 2) to eliminate discontinuity due to missing values; fold-change values that were less than one were replaced by the negative of their inverse. Percentage coefficient of variation (%CV) was also measured for each protein based on mean and standard deviation of NSAF values. Besides having an FDR <0.05, two further filters (fold-change >1.3 or <−1.3 and %CV lower than the percentage change corresponding to the fold change) were established to define a significantly differential protein. Data were parsed using in-house scripts, and graphs were generated using Microsoft Excel. Network and pathway analysis were carried out using Ingenuity Pathways Analysis (IPA, version 9.0, Ingenuity Systems, Redwood City, CA), considering both direct and indirect relationships and using protein fold-change >1.3 (or <−1.3) and FDR <0.05 as cut-off.

## Results

### Differential label-free proteomic analysis

Proteomic analysis allowed the overall identification of 903 proteins, of which 756 in the control (untreated MM cells) and 630 in the 10 μM D6 treated MM cells (Additional file 1: Table S1 and Additional file 2: Table S2, respectively).

**Fig. 2:**
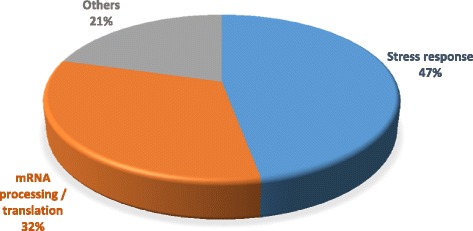
Major functional classification of differentially abundant proteins. The graph represents the major functional groups comprising the 34 proteins identified by differential analysis, as listed in Table 3

A label-free, spectral counting approach was employed to identify differentially expressed proteins, and thus to focus on changes in protein expression induced by D6 treatments. Using FDR <0.05 as significance threshold and fold-change >1.3 or <−1.3 as differential abundance threshold, 18 and 16 proteins were found as up- and down-regulated by D6 treatments, respectively (Tables 1 and 2). Most of these 34 selected proteins could be classified into two major groups according to the putative kind of cell process they are related (Fig. 2), as listed in Table 3. The first group comprises about half of the modulated proteins (47 %), all involved in cell stress response, namely: heat shock proteins (HSP)/chaperonins and other proteins involved in endoplasmic reticulum (ER) protein processing [22, 23]; ubiquitin, related to protein degradation process [24]; histones H2AX and H2AZ both involved in DNA repair [25–27], and others possibly playing a role in stress response [28–30]. Moreover, the YWHAE and YWHAZ isoforms of the 14-3-3 protein, widely involved in signal transduction, could be included in this functional group because of their activity in regulation of cell proliferation and survival as a response to stress stimuli [31]. Most of these proteins showed to be upregulated by D6 treatment indicating activation of cell defense processes. The second group representing one-third of the differentially abundant proteins includes proteins involved in the mRNA processing and protein translation machinery [32–39], mostly showing to be downregulated. The decrease in abundance of proteins within this major functional group suggests a reduction of both translation and protein synthesis in the cells, which can be interpreted as a sign of cell growth arrest.

**Table 1:** Upregulated proteins

Accession	Gene symbol	Protein name	Fold-change treated/control	FDR
**P0CG48**	**UBC**	**Polyubiquitin-C**	**6.32**	**0.00000**
P61604	HSPE1	10 kDa heat shock protein, mitochondrial	5.82	0.00001
**P08107**	**HSPA1/HSP70**	**Heat shock 70 kDa protein 1A/1B**	**4.97**	**0.00007**
P62318	SNRPD3	Small nuclear ribonucleoprotein Sm D3	4.20	0.00325
**P25685**	**DNAJB1**	**DnaJ homolog subfamily B member 1**	**3.72**	**0.00706**
P23396	RPS3	40S ribosomal protein S3	3.72	0.00325
**Q92598**	**HSPH1**	**Heat shock protein 105 kDa**	**3.51**	**0.00080**
P04264	KRT1	Keratin, type II cytoskeletal 1	3.42	0.04177
**P62258**	**YWHAE**	**14-3-3 protein epsilon**	**3.38**	**0.03245**
P63244	GNB2L1	Guanine nucleotide-binding protein subunit beta-2-like 1	3.05	0.04582
P63104	YWHAZ	14-3-3 protein zeta/delta	2.89	0.00730
**P34931**	**HSPA1L**	**Heat shock 70 kDa protein 1-like**	**2.82**	**0.00371**
P07195	LDHB	L-lactate dehydrogenase B chain	2.70	0.03245
**P17066**	**HSPA6**	**Heat shock 70 kDa protein 6**	**2.58**	**0.02396**
P68363	TUBA1B	Tubulin alpha-1B chain	2.39	0.01570
**P48741**	**HSPA7**	**Putative heat shock 70 kDa protein 7**	**2.36**	**0.04329**
P04792	HSPB1	Heat shock protein beta-1	2.27	0.01149
P62805	HIST1H4A	Histone H4	1.52	0.00054

The table lists the proteins that showed to be upregulated (fold-change >1.3) in LB24Dagi MM cells after 24 h of 10 μM D6 treatments (FDR <0.05). Proteins modulated also at the gene expression level are shown in bold characters [10]

**Table 2:** Downregulated proteins

Accession	Gene symbol	Protein	fold-change treated/control	FDR
P83731	RPL24	60S ribosomal protein L24	−4.14	0.00054
P61254	RPL26	60S ribosomal protein L26	−3.63	0.02751
P52272	HNRNPM	Heterogeneous nuclear ribonucleoprotein M	−3.24	0.00004
P18621	RPL17	60S ribosomal protein L17	−3.11	0.02499
**O60506**	**SYNCRIP**	**Heterogeneous nuclear ribonucleoprotein Q**	**−2.89**	**0.04096**
P62280	RPS11	40S ribosomal protein S11	−2.73	0.00944
P23284	PPIB	Peptidyl-prolyl cis-trans isomerase B	−2.41	0.02471
P16104	H2AFX	Histone H2AX	−2.36	0.00054
P13667	PDIA4	Protein disulfide-isomerase A4	−2.05	0.04384
P16104	H2AFZ	Histone H2A.Z	−1.99	0.00109
**Q93077**	**HIST1H2AC**	**Histone H2A type 1-C**	**−1.92**	**0.00096**
P04908	HIST1H2AB	Histone H2A type 1-B/E	−1.77	0.00165
P20671	HIST1H2AD	Histone H2A type 1-D	−1.75	0.01570
Q92841	DDX17	Probable ATP-dependent RNA helicase DDX17	−1.63	0.00109
Q9UKM9	RALY	RNA-binding protein Raly	−1.47	0.00531
Q9Y6E2	BZW2	Basic leucine zipper and W2 domain-containing protein 2	−1.31	0.00531

The table lists the proteins that showed to be downregulated (fold-change <−1.3) in LB24Dagi MM cells after 24 h of 10 μM D6 treatments (FDR <0.05). Proteins modulated also at the gene expression level are shown in bold characters [10]

**Table 3:** Major functional classification of differentially abundant proteins

	Stress response		mRNA processing/translation		Others
1	UBC [24] ↑	1	SNRPD3 [33] ↑	1	KRT1 [40] ↑
2	HSPE1 [22] ↑	2	GNB2L1 [36] ↑	2	LDHB [43] ↑
3	HSPA1/HSP70 [22] ↑	3	RPL24 [32] ↓	3	TUBA1B [41] ↑
4	DnaJ B 1 [22] ↑	4	RPL26 [32] ↓	4	HIST1H4A [42] ↑
5	RPS3 [28] ↑	5	HNRNPM [34] ↓	5	HIST1H2AC [42] ↓
6	HSPH1 [22] ↑	6	RPL17 [32] ↓	6	HIST1H2AB [42] ↓
7	YWHAE [31] ↑	7	SYNCRIP [35] ↓	7	HIST1H2AD [42] ↓
8	YWHAZ [31] ↑	8	RPS11 [32] ↓		
9	HSPA1L [22] ↑	9	DDX17 [37] ↓		
10	HSPA6 [22] ↑	10	RALY [38] ↓		
11	HSPA7 (HSP90AA1) [22] ↑	11	BZW2 [39] ↓		
12	HSPB1 [22] ↑				
13	PPIB [29] ↓				
14	H2AFX [26] ↓				
15	PDIA4 [30] ↓				
16	H2AFZ [27] ↓				

Proteins in the table are grouped according to their putative role in our model. Each protein is identified by the corresponding gene symbol. Up- or down-facing arrows indicates up- or down-regulated proteins, respectively

The remaining seven regulated proteins exert various functions: Keratin 1 (KRT1) and Tubulin α-1b (TUBA1B) represent structural proteins [40, 41] while HIST1H4A, HIST1H2AB HIST1H2AC, HIST1H2AD all belong to the replication-dependent histone family [42]; finally, LDHB is a lactate dehydrogenase subunit participating in pyruvate to lactate interconversion [43].

On general scale, the protein expression changes partially reflect the biological effects previously observed on D6-treated LB24Dagi cells: activation of cell stress responses accompanied by arrest of both cell growth and cell functions [9, 10].

### Functional annotation and pathway analysis

The differential proteomic profiles obtained upon comparison of D6 treated and untreated MM cells were analyzed by the Ingenuity Pathway Analysis (IPA) software, focusing on statistically differential proteins (FDR <0.05).

Table 4 lists the most significant biological functions and diseases that appeared to be influenced by D6 activity, based on the protein abundance changes induced by D6 treatments. Among the top bio-functions related to the action of D6 on LB cells, we found several cell processes involved in cell functions, maintenance, and survival, including gene expression and protein synthesis, as well as cell cycle, cell death and cancer (Table 4), thus demonstrating D6 interference with key cell activities.

**Table 4:** IPA top bio functions and diseases

	Top bio functions and diseases	*p-value*	# molecules
**1**	**Cellular Compromise**	**2.26E-06 - 4.58E-02**	**7**
**2**	**Cellular Function and Maintenance**	**2.26E-06 - 4.72E-02**	**8**
**3**	**Gene Expression**	**3.39E-06 - 2.74E-02**	**14**
**4**	**Protein Synthesis**	**4.88E-06 - 1.74E-02**	**10**
5	Drug Metabolism	7.40E-05 - 3.31E-02	4
6	Small Molecule Biochemistry	7.40E-05 - 3.31E-02	8
**7**	**Dermatological Diseases and Conditions**	**9.02E-05 - 8.75E-03**	**7**
**8**	**Cell Cycle**	**1.45E-04 - 3.88E-02**	**6**
9	Neurological Disease	1.49E-04 - 1.61E-02	10
**10**	**Post-Translational Modification**	**3.36E-04 - 3.74E-02**	**6**
11	Protein Folding	3.36E-04 - 3.48E-02	3
12	Cell Morphology	5.78E-04 - 4.30E-02	9
**13**	**Cellular Assembly and Organization**	**5.78E-04 - 4.58E-02**	**8**
**14**	**Cell Death and Survival**	**8.34E-04 - 4.86E-02**	**13**
15	Psychological Disorders	8.35E-04 - 3.14E-02	6
**16**	**Skeletal and Muscular Disorders**	**8.35E-04 - 4.18E-02**	**12**
**17**	**Cellular Development**	**1.31E-03 - 3.45E-02**	**6**
18	Skeletal and Muscular System Development and Function	1.31E-03 - 1.02E-02	4
**19**	**Tissue Development**	**1.31E-03 - 4.72E-02**	**7**
**20**	**Cancer**	**1.46E-03 - 5.00E-02**	**19**
21	Cardiovascular Disease	1.46E-03 - 2.61E-02	5
**22**	**DNA Replication, Recombination, and Repair**	**1.46E-03 - 4.86E-02**	**6**
23	Developmental Disorder	1.46E-03 - 2.89E-02	7
24	Embryonic Development	1.46E-03 - 3.60E-02	5
25	Hematological Disease	1.46E-03 - 4.16E-02	9
26	Hereditary Disorder	1.46E-03 - 2.89E-02	6
**27**	**Immunological Disease**	**1.46E-03 - 4.18E-02**	**12**
28	Nervous System Development and Function	1.46E-03 - 4.86E-02	4
29	Organ Morphology	1.46E-03 - 4.16E-02	4
30	Organismal Development	1.46E-03 - 4.16E-02	4
31	Organismal Injury and Abnormalities	1.46E-03 - 5.00E-02	21
32	Respiratory Disease	1.46E-03 - 4.58E-02	5
**33**	**Tissue Morphology**	**1.46E-03 - 4.72E-02**	**4**
**34**	**Reproductive System Disease**	**1.68E-03 - 5.00E-02**	**11**
**35**	**RNA Post-Transcriptional Modification**	**1.77E-03 - 2.78E-02**	**4**

The table lists the biological functions and diseases identified by IPA as the most significantly influenced by D6 activity (*p value* ≤0.005, >2 identified molecules). *p-values* are reported as range of values referred to the several sub-groups classified by IPA into each bio-functions and disease major group. Bio-functions and diseases found statistically significant also by the gene expression profile analysis (*p value* ≤0.001) are shown in bold characters [10]

Table 5 reports the canonical pathways that appeared significantly influenced by changes in protein abundance subsequent to the D6 treatment in MM cells. These mostly reflect the main responses of the cell to D6 already highlighted by expression profiling [10]: involvement of both cellular stress response (pathways 1, 2, 4, 5, 8) and cell growth and proliferation related processes (pathways 3, 6–11).

**Table 5:** IPA top canonical pathways

	Ingenuity canonical pathways	-log(B-H *p*-value)	Molecules
**1**	**Protein Ubiquitination Pathway**	**5,52E00**	**HSPB1,HSPA1L,DNAJB1,HSPH1,HSPA6,HSPE1,UBC**
**2**	**Aldosterone Signaling in Epithelial Cells**	**5,52E00**	**HSPB1,HSPA1L,DNAJB1,HSPH1,HSPA6,HSPE1**
3	EIF2 Signaling	5,19E00	RPL17,RPL24,RPS27,RPS11,RPL26,RPS3
4	Huntington’s Disease Signaling	3,43E00	HSPA1L,DNAJB1,GNB2L1,HSPA6,UBC
5	Unfolded protein response	2,98E00	HSPA1L,HSPH1,HSPA6
6	14-3-3-mediated Signaling	2,06E00	TUBA1B,YWHAE,YWHAZ
7	Regulation of eIF4 and p70S6K Signaling	1,85E00	RPS27,RPS11,RPS3
**8**	**Cell Cycle: G2/M DNA Damage Checkpoint Regulation**	**1,65E00**	**YWHAE,YWHAZ**
9	mTOR Signaling	1,65E00	RPS27,RPS11,RPS3
10	Myc Mediated Apoptosis Signaling	1,59E00	YWHAE,YWHAZ
11	ERK5 Signaling	1,56E00	YWHAE,YWHAZ

The table lists the canonical pathways identified by IPA as the most significantly involved in D6 biological effects (−logp-value ≤1.5 after multiple testing correction according to Benjamini Hochberg). Pathways found statistically significant also by the gene expression profile analysis (*p value* ≤0.001) are shown in bold characters [10]

**Fig. 3:**
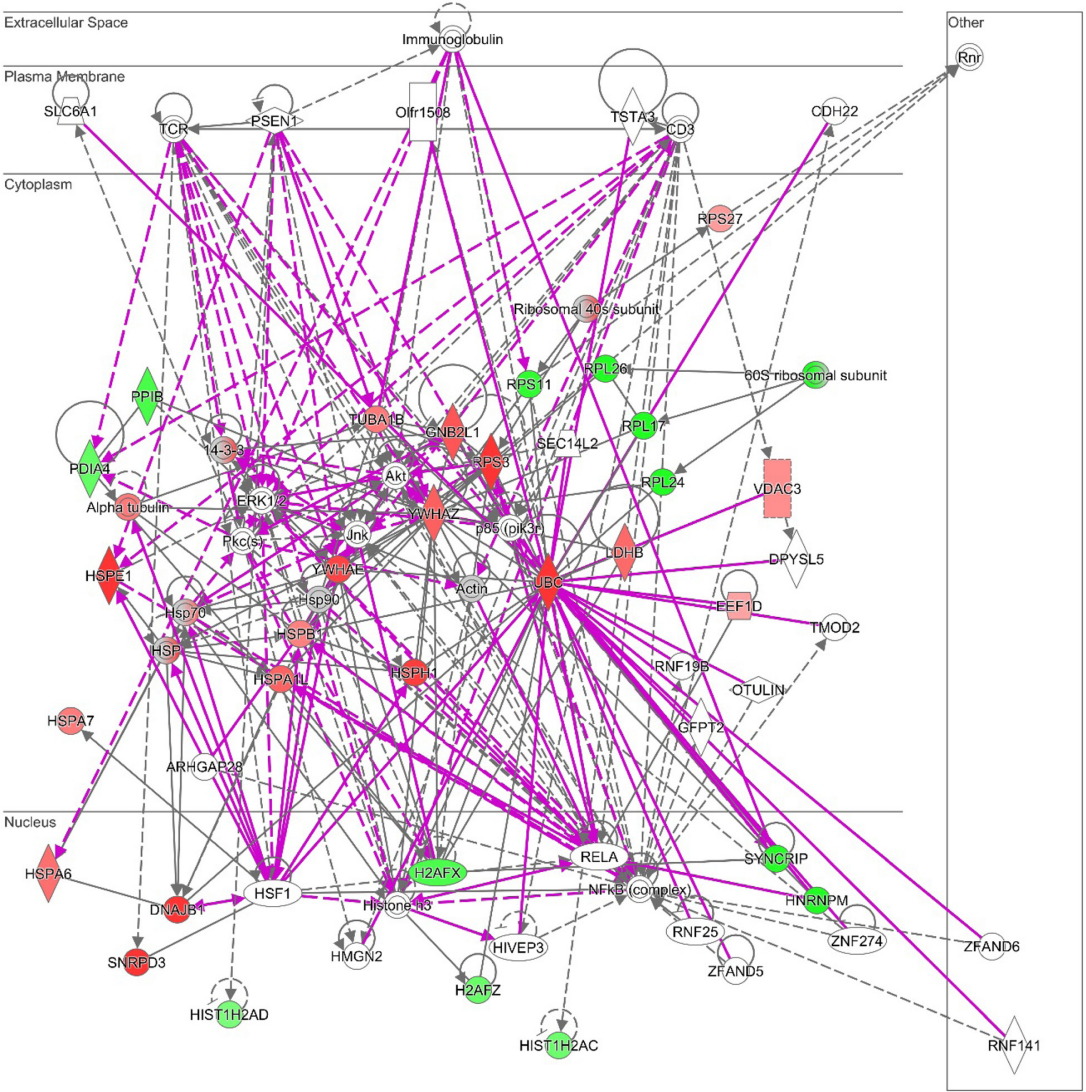
D6 activity molecular network. The diagram shows merging of network 1 and 2 and schematizes the predicted distribution of the protein networks members according to their cellular localization. Up-regulated proteins are represented in red gradations, while down-regulated ones in green

Table 6 lists the two major protein networks related to D6 treatment, according to IPA analysis. Again, they show the interference of D6 activity with important cellular processes. The first one evidences the intersections between the two most represented groups of modulated proteins listed in Table 3 (stress response and mRNA processing/translation) with all the correlations elapsing among molecules involved in RNA processing and protein synthesis and molecules involved in cell response to injuries and abnormalities. The second network underlines the involvement of cell signaling and cell-to-cell interaction. Figure 3 shows the intersections among the two networks and the predicted distribution of the protein network members according to their cellular localization.

**Table 6:** IPA top networks

ID	Molecules in network	Score	Focus molecules	Top diseases and functions
**1**	**14-3-3**, 60S ribosomal subunit, Actin, **Alpha tubulin**, **DNAJB1**, ERK1/2, **GNB2L1**, **H2AFX**, **H2AFZ**, Histone h3, **HNRNPM**, HSP, **HSP70**, Hsp90, **HSPA6**, **HSPA1L**, **HSPB1**, **HSPE1**, **HSPH1**, **LDHB**, **PDIA4**, **PPIB**, 40S ribosomal subunit, Rnr, **RPL17**, **RPL24**, **RPL26**, **RPS3**, **RPS11**, RPS27, **SYNCRIP**, **TUBA1B**, **UBC**, **YWHAE**, **YWHAZ**	67	26	Protein Synthesis, Cellular Compromise, Cellular Function and Maintenance
**2**	Akt, ARHGAP28, CD3, CDH22, DPYSL5, **EEF1D**, GFPT2, **HIST1H2AC**, **HIST1H2AD**, HIVEP3, HMGN2, HSF1, **HSPA7**, Immunoglobulin, Jnk, NFkB (complex), Olfr1508, OTULIN, p85 (pik3r), Pkc(s), PSEN1, RELA, RNF25, RNF141, RNF19B, SEC14L2, SLC6A1, **SNRPD3**, TCR, TMOD2, TSTA3, **VDAC3**, ZFAND5, ZFAND6, ZNF274	12	6	Cell-To-Cell Signaling and Interaction, Nervous System Development and Function, Cell Signaling

The table lists the two major protein networks (score >10) related to D6 activity on MM cells identified by IPA analysis. Networks are scored based on the number of dataset molecules they contain (“Focus molecules” column). The higher the score, the lower the probability of finding the observed number of dataset molecules in a given network by random chance. Molecules observed in this study are reported in bold characters in the column “molecules in network”

## Discussion

The analysis of the protein complement expressed by a cell in a given moment or condition gathers information on the real potential expressed by the genome. In fact, gene expression analysis, although offering the advantage of a wider coverage, is biased by the vast plethora of variables that come into play after gene transcription, such as the different half lives of transcripts, their transcriptional efficiency, as well as the different half lives of proteins within the cell. By directly measuring protein abundances, differential proteomics has the advantage of providing a more realistic picture of the cell response to a given stimulus, although the number of proteins that can be evaluated is often lower than the one obtained in a transcriptomic study. In a recent paper, we described the changes induced by D6 treatments in the gene expression profile of LB24Dagi MM cells [10]. In order to integrate and validate those findings, a differential proteomics study was carried out, and it is described here.

When comparing the list of proteins modified by the D6 treatment with the results of the microarray gene expression profiling, only 10 direct correspondences were observed: upregulation of six HSPs, Ubiquitin, and YWHAE, and downregulation of SYNCRIP and HIST1H2AC (bolded in Tables 1 and 2). Despite the short list of overlapping IDs between microarray and proteome differential analyses, pathways and network analysis using IPA software pointed out the alteration of similar biological functions and molecular pathways in D6 treated MM cells (see Tables 4 and 5). Moreover, the 10 common IDs observed between the two approaches can be well framed in the global scenario resulting from IPA bio-functions and pathways analyses, as discussed below. In fact, it is becoming evident that transcriptomics and proteomics data do not have often a clear overlap in protein identities, but these can share the same functional content, that can be evidenced by extracting information in terms of protein interaction networks and pathways [44, 45].

The most significant result of the differential proteomic analysis is a confirmation of a stress induced cell response, as previously observed at the gene expression level [10]. Indeed, current pathway analysis results indicate the strong involvement of stress induced molecular pathways (Table 5: pathways 1, 2, 4, 5 and 8), three of which (1, 2, 8) were also found to be significantly modified in our previous gene expression profiling [10]. The up-regulation of eight proteins belonging to the HSPs family (six in common with the gene expression analysis) is consistent with this observation; in addition, it suggests the involvement of protein processing mechanisms in the endoplasmic reticulum and stimulation of apoptotic pathways as one of the major D6 effects on MM cells [22, 23]. Moreover, proteins PPIB and PDIA4, also involved in the protein processing inside the endoplasmic reticulum [29, 30], appeared to be down-regulated (Table 2), thus confirming the influence of D6 on this process. Abundance of ubiquitin and the significance of pathway 1 (*Protein ubiquitination*) involvement (Table 5) could be related to an increase in protein degradation processes due to severe protein damage in stressed cells committed to apoptosis, beside of the central role of the ubiquitination process in cell cycle regulation [46]. It has been largely demonstrated that an intense crosstalk exists between the apoptotic pathways and the ubiquitin and proteasome system whose function in apoptosis appears to be very complex (reviewed in [24]).

The reduced levels of H2AX and H2AZ, histone H2A variants involved in DNA repair and regulation of transcription [25–27], instead could be related to a decrease of transcription activity and to the lack of DNA repairing needs in apoptotic cells.

Such results highlight the activation of a complex anti-stress molecular network as a cell response to D6 activity. In order to maintain a stable intracellular environment, cells utilize complex and specialized defense systems against various type of external perturbations, activating many adaptive mechanisms that appear to operate through gene regulatory networks [47]. This complex molecular response certainly gives rise to alteration of several processes deeply modifying cell behavior and producing changing in protein expression, as we observed in our experimental model. The abundance of YWHAE and YWHAZ proteins (Table 1), isoforms of the 14-3-3 protein that regulate multiple cellular functions via interactions with phosphorylated partners, could be an aspect of such a response [48]. Indeed, 14-3-3 proteins act as integrators of various signals that influence cell fate decisions and tumorigenesis, regulating apoptosis, mitogenic and stress signaling, and cell-cycle progression [31]. In our system, their up-regulation is associated with activation of several pathways involved in cell cycle control and apoptosis (see Table 5, pathways 6 -14-3-3 mediated signaling, 8 -Cell Cycle: G2/M DNA Damage Checkpoint Regulation, 10 -Myc Mediated Apoptosis Signaling, 11 -ERK5 signaling), thus attesting the activation of a complex molecular signaling network. Figure 3 shows the diagram elaborated by IPA, which schematizes the molecular network created by D6 activity in our MM in vitro model.

The interference of D6 activity with several intracellular processes regulating cell proliferation and survival is well evidenced by the results of IPA bio-functions analysis (Table 4). Indeed, bio-functions such as *Gene expression*, *Protein synthesis*, *Cell cycle*, *Cell Death and Survival* were found significantly influenced by D6 induced proteomic changes (Table 4).

These results are tightly consistent with those presented in our previous papers, which pointed out the involvement of similar bio-functions and molecular pathways, and demonstrated that D6 causes a cell cycle arrest at the G2/M phase and drives cells to apoptosis [9, 10]. Both cell cycle arrest and apoptosis are obviously accompanied by a decrease or even by a complete block of protein synthesis [49], which in the present study is evidenced by a decreased level of several proteins participating in mRNA processing and in translation. In fact, three HNRNPs (HNRNPM, SYNCRIP, RALY) and the RNA helicase DDX17, all involved in splicing, polyadenylation and other aspects of mRNA maturation and transport [34, 35, 37, 38] were found to be downregulated (Table 2). Moreover, four ribosomal proteins (RPL24, RPL17, RPL26, RPS11), components of either 60S or 40S ribosomal subunits [32], and the translational regulator BZW2 [39], were all detected as decreased in D6 treated cells, thus confirming a downregulation of the protein translation process. Instead, RPS3 [28] and GNB2L [36], both components of 40S ribosome subunit, as well as SNRPD3, a component of the spliceosome [33], appeared to be up-regulated, probably just because of a general deregulation of survival processes.

The interference of D6 activity with translation and protein synthesis process is also depicted by the pathway analysis results (Table 5), where pathways three (EIF2 signaling), seven (eIF4 signaling) and nine (mTOR signaling), are all related to translation and protein synthesis [50, 51].

Taken together, the data obtained as a result of the differential proteomic analysis in D6 treated melanoma cells generally confirm our previous findings on gene expression profiling in the same conditions, even if we did not find a precise correlation among the specific molecules detected with the two approaches. In fact, it should be noted that, apart from HSPs and few others proteins related to cell stress response, none of the proteins corresponding to the genes whose expression was strongly modulated by D6 was found to be significantly modulated at the protein level. Such a discrepancy might be explained by differences in specificity and sensitivity between the two approaches, as well as by the wealth of complex molecular mechanisms underlying mRNA and protein degradation and turnover. In order to go deep into this aspect, specific protein expression changes highlighted in this paper are currently being investigated through western blot analysis and will be included in a future publication.

## Conclusions

In conclusion, the most significant differentially modulated proteins following D6 treatment in cultured melanoma cells are represented by proteins involved in stress cell response (up modulated) and in translation (down modulated). HSPs are the mainly affected protein family. This is consistent with our previous findings and with the biological effects of D6 on MM cells; in fact, HSPs have a protective function in stress conditions but also an essential role in apoptosis regulation, which is one of the D6 mechanisms underlying tumor growth inhibition. Bio-functions and pathways analyses by IPA pointed out the involvement of cellular processes strictly associated to stress and damage cell response, which, in turn, activate a molecular network involving decrease of protein synthesis and reduction of vital cell activity. These dramatic changes in the cellular environment finally appeared responsible of the cell proliferation arrest and apoptosis observed in our MM model after D6 treatments. Taken together, our results are in agreement with the ones obtained with the gene expression profile analysis, thus confirming the efficacy of D6 as a promising anti-melanoma agent. In this sense, its large spectrum activity makes it a good candidate to be considered in combination therapies with targeted agents, offering a therapeutic option toward the reduction of drug resistance and disease recurrence.

### Ethics approval and consent to participate

For all melanoma cell lines, tumor tissues were processed after obtaining the patient’s informed consent and according to the approval by the Ethics Committee at the “Istituto Dermopatico dell’Immacolata” in Rome, Italy.

### Consent for publication

Not applicable.

### Availability of data and materials

The datasets supporting the conclusions of this article are included within the article (and its additional files).

## Additional files

Additional file 1: Table S1. Control MM cells identified proteins. Complete list of proteins identified from the untreated malignant melanoma cells (control). Normalized spectral abundance factor (NSAF) values are reported for each protein. (XLS 160 kb) Additional file 2: Table S2. D6 treated MM cells identified proteins. Complete list of proteins identified from the 10 μM D6 treated malignant melanoma cells (treated). Normalized spectral abundance factor (NSAF) values are reported for each protein. (XLS 142 kb)

## Abbreviations

%CV: percentage coefficient of variation
CF: correction factor
DMSO: dimethyl sulfoxide
ER: endoplasmic reticulum
FDR: false discovery rate
HSP: heat shock protein
IPA: Ingenuity Pathway Analysis
LB: LB24Dagi melanoma cell line
MM: malignant melanoma
NSAF: normalized spectral abundance factor
SAF: spectral abundance factor
SpC: spectral count
